# Using daily diagnostic quality images to validate planning margins for prostate interfractional variations

**DOI:** 10.1120/jacmp.v17i3.5923

**Published:** 2016-05-08

**Authors:** Wen Li, Andrew Vassil, Andrew Godley, Lama Muhieddine Mossolly, Qingyang Shang, Ping Xia

**Affiliations:** ^1^ Department of Radiation Oncology Cleveland Clinic Cleveland OH USA

**Keywords:** prostate cancer, interfraction, planning margins, diagnostic image

## Abstract

The purpose of this study is to use the same diagnostic‐quality verification and planning CTs to validate planning margin account for residual interfractional variations with image‐guided soft tissue alignment of the prostate. For nine prostate cancer patients treated with IMRT to 78 Gy in 39 fractions, daily verification CT‐on‐rails images of the first seven and last seven fractions (n=126) were retrospectively selected for this study. On these images, prostate, bladder, and rectum were delineated by the same attending physician. Clinical plans were created with a margin of 8 mm except for 5 mm posteriorly, referred to as 8/5 mm. Three additional plans were created for each patient with the margins of 6/4 mm, 4/2 mm, and 2 mm uniform. These plans were subsequently applied to daily images and radiation doses were recalculated. The isocenters of these plans were placed according to clinical online shifts, which were based on soft tissue alignment to the prostate. Retrospective offline shifts by aligning prostate contours were compared to online shifts. The resultant daily target dose was analyzed using $D_{99}$, the percentage of the prescription dose received by 99% of CTV. The percent of bladder volume receiving $65\;\text{Gy}\;(V_{65\text{Gy}})$ and rectum $V_{70\text{Gy}}$ were also analyzed. After interfractional correction, using CTV $D_{99}>97\%$% criteria, 8/5 mm, 6/4 mm, 4/2 mm, and 2 mm planning margins met the CTV dose coverage in 95%, 91%, 65%, and 53% of the 126 fractions with online shifts, and 99%, 98%, 85%, and 68% with offline shifts. The rectum $V_{70\text{Gy}}$ and bladder $V_{65\text{Gy}}$ were significantly decreased at each level of margin reduction (p<0.05). With daily diagnostic quality imaging‐guidance, the interfractional planning margin may be reduced from 8/5 mm to 6/4 mm. The residual interfractional uncertainties most likely stem from prostate rotation and deformation.

PACS number(s): 87.53.‐j

## I. INTRODUCTION

Precise target localization is required for intensity‐modulated radiation therapy (IMRT) but is challenging for prostate cancer treatment due to interfractional patient setup and anatomic variations. The clinical target volume (CTV) is expanded to a planning target volume (PTV) to ensure adequate target dose coverage to account for setup uncertainties, interfractional and intrafractional organ motions. A large PTV expansion adversely affects the radiation dose to the nearby organs at risk (OAR). Data from multiple centers associate high rectum and bladder doses with higher incidences of late toxicities.^(1)^ A meta‐analysis of over 10,000 patients treated using IMRT for prostate cancer showed a grade 2 or above late gastrointestinal (GI) toxicity of 15% and genitourinary (GU) toxicity of 17%.^(2)^ This suggests PTV reduction should decrease the toxicity. Because of the emergence of new image‐guided radiation therapy (IGRT) techniques, PTV reduction deserves further examination.^(3)^ This is particularly important for stereotactic body radiation therapy (SBRT)^(4)^ as the effect of interfraction dose variation may be greater with fewer fractions.^(5)^

The main clinical practice to compensate for the interfractional variations is to reposition patients prior to treatment according to the registration of the daily image and planning image. Both implanted markers and soft tissue are effective for the image registration.^6^, ^7^, ^8^ Marker‐based registration, however, requires an invasive implantation procedure, which may cause discomfort, bleeding, and infection.^(9)^ Furthermore, marker‐based registration provides little information about the rotation or deformation of the prostate, and marker migration may pose issues with accurate target localization.^(10)^ It can be difficult to localize the seminal vesicles and to detect changes in the surrounding normal anatomy.^(11)^

While kV‐CBCT is known to suffer from suboptimal soft tissue contrast and artifacts,^(12)^ an in‐room, CT‐on‐rails system has been recently developed to provide daily images with diagnostic quality soft tissue contrast. In a recent study using CT‐on‐rails guided IMRT, an 8/5 mm planning margin was shown to provide an average CTV $D_{100}$ of 98.1% with respect to the original plan.^(13)^ However, whether the 8/5 mm planning margin for interfractional variations can be reduced without compromising target coverage remains to be explored. In the era of SBRT, with IGRT image quality the same as planning CTs, one may expect near zero interfractional planning margins.

In the present study, the daily dose profile was assessed with soft tissue‐based IGRT from CT‐on‐rails for the clinical margin of 8/5 mm and three additional retrospectively created margins of 6/4 mm, 4/2 mm, and 2 mm uniform. For each margin, daily dose calculation was performed with the clinical daily treatment isocenters. To explore the margin required for IGRT with best possible translational shifts, daily dose profiles were also calculated with retrospective offline shifts that aligned the daily and planning prostate contours.

## II. MATERIALS AND METHODS

Nine consecutive patients treated with IMRT to 78 Gy in 39 fractions for prostate cancer were included in this analysis. To match similar number of fractions in SBRT, we chose to analyze daily verification images from the first seven fractions. As patients typically show greater volume changes in the ending fractions of the treatment course,^(13)^ the daily verification CT images of the last seven fractions were also separately analyzed. Patients were instructed to have a full bladder and empty rectum prior to treatment. The same condition was applied for simulation with the patients given 500 cc of water to drink one hour before the simulation. During treatment, each patient was positioned supine with an indexed knee cushion, feet banded, hands holding a foam ring.

For each patient, daily verification CT images were acquired using a CT‐on‐rails system (Siemens Medical Solutions USA, Concord, CA). The CT unit (Siemens, Somato, Sensation Open, 24 slices) is an integrated part of the linear accelerator (Siemens, ARTISTE). The same CT unit was also used to acquire planning CTs. The planning CT and verification CT for each patient were acquired with the same acquisition protocol, typically using 120 kVp, 260 mAs with a field of view of 50.0 cm and a slice thickness of 3 mm.

After acquisition, the verification CT was automatically registered with the planning CT based on bony structures using IGRT software (Syngo, Siemens) and then manually adjusted based on the soft tissue of the prostate by the therapists. The resultant treatment isocenter shifts were applied before each treatment. The prostate, rectum, and bladder on the verification CTs were retrospectively delineated by the same attending physician who contoured the initial treatment plans. The daily volumes of the three organs were calculated and compared with those in the planning CT.

To explore the margin required with best possible IGRT shifts for the scenario of SBRT treatments with direct supervision of the attending physician and physicist, an offline alignment for each verification CT was also performed using MIM software (MIM Software Inc., Cleveland, OH). The contour‐based registration was performed as follows: a) found the center of the mass (COM) of each contour; b) aligned two COMs via the point alignment method; c) manually adjusted the alignment by visual inspection. We found this method rendered the best consistency and the greatest similarity between daily and planning prostate volume.

All patient plans were created in Pinnacle 9.0 (Philips Medical Systems, Fitchburg, WI) using IMRT with the typical seven beam angles $-0^{\circ }$, 55°, 90°, 135°, 225°, 270°, and 315° – following IEC convention. The PTV was an 8 mm expansion of the prostate (CTV) except for 5 mm posteriorly (8/5 mm). A dose of 78 Gy in 39 fractions was prescribed. For this study, three additional treatment plans were retrospectively generated for each patient with planning margins of 6/4 mm, 4/2 mm, and 2 mm uniform. The IMRT plans created with these margins used the same beam arrangements and same total number of segments as the clinical plans. The optimization constraints and the prescription isodose lines were adjusted to ensure that at least 95% of the new PTV and at least 99% of the CTV received the prescription dose. The clinical and reduced margin plans were subsequently applied to each of the 126 verification CTs and doses were recalculated with isocenter placed according to the clinical corrections applied to the patients (online shifts), resulting in a total of 504 verification plans. For each verification plan, another dose calculation was performed with the isocenter placed according to the retrospective offline shifts. Therefore, a total of 1,008 daily dose distributions were assessed.

### A. Dosimetric endpoints and statistic analysis

The endpoint for target dose coverage was chosen as the percentage of the daily prescription dose received by 99% of the CTV ($D_{99}$). Although the plan acceptance criteria was $D_{99}>99\%$ for the entire treatment, we analyzed both daily CTV $D_{99}>99\%$,>97%,>95%,>90%, and>80% for all four planning margins. The endpoint for the rectum is the percentage of volume receiving 70 Gy over the entire treatment course, $V_{70\text{Gy}}$. Daily rectal dose with an equivalent of $V_{70\text{Gy}}>30\%$,>25%,>20%, and>10% was analyzed. $V_{70\text{Gy}}>20\%$ has been consistently associated with rectal bleeding or Grade ≥ 2 late rectal toxicity.^(1)^ The endpoint of bladder was chosen as $V_{65\text{Gy}}$. Daily bladder dose with an equivalent of $V_{65\text{Gy}}>30\%$,>20%, and>10% was analyzed.

Descriptive statistics were used for data analysis. Two‐tailed unpaired Student's *t*‐tests were used to compare the dosimetric endpoints, the change of daily organ volumes from the planning volumes, and the daily IGRT shifts for the first seven and the last seven fractions. Two‐tailed paired Student's *t*‐tests were used to compare the endpoints from different margin reduction plans and to compare the endpoints from the online and offline shifts. Statistical significance was assigned at p<0.05.

## III. RESULTS

### A. Daily volume variations

**Figure 1:**
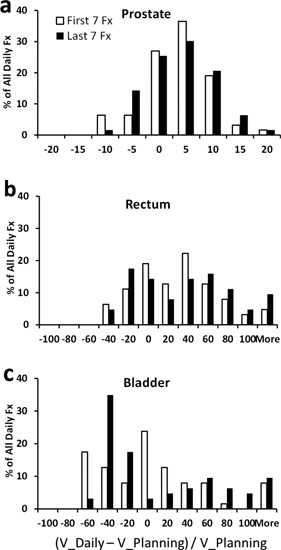
Histogram of the percentage changes of daily volumes (V_Daily) from the corresponding volume in the planning CTs (V_Planning) for (a) prostate, (b) rectum, and (c) bladder.

Figure 1 shows the interfractional volume changes of the prostate, rectum, and bladder. The prostate volume in the planning CTs ranged from 19.8 cc to 75.9 cc, with an average of 49.2 cc ± 17.6 cc. The average daily prostate volume of the 126 fractions were 1.3% ± 6.0% (range: −14.9% to 16.0%) greater than the prostate volume from the planning CTs (Fig. 1(a)). The average daily rectum volume was 24.3%±47.3% greater than the rectum volume from the planning CTs, suggesting that the patients did not strictly comply with the instructions to maintain an empty rectum (Fig. 1(b)). The daily rectum volume also shows a relatively large fluctuation (−50.4% to 47.3%) in reference to the rectum volume from the planning CTs (Fig. 1(b)). The daily bladder volume also greatly fluctuated from the planned volume (range: −79.7% to 203%) on a daily basis, although on average it is only 0.1%±58.1% less from the planning volume (Fig. 1(c)).

In reference to the volumes from planning CTs, the average daily volume changes of the last seven fractions were comparable to those of the first seven fractions for prostate (1.8%±5.9% and 0.7%±6.2%, p=0.30), rectum (27.7%±50.4% and 21.0%±44.2%, p=0.43), and bladder (2.4%±58.8% and −2.6%±57.7%, p=0.62). However, more daily fractions showed large (>20%) volume changes in the last seven fractions than in the first seven fractions for the rectum (74.6% and 55.8%) and the bladder (60.2% and 57.0%). For the prostate, the volume fluctuation level was similar, with 1.6% of fractions showing >15% volume changes for both first seven and last seven fractions.

### B. Online and offline shifts

**Figure 2:**
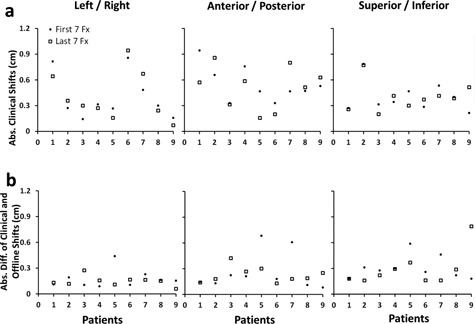
(a) Magnitude of the clinical online IGRT shifts. (b) Magnitude of the difference between the online and offline IGRT shifts.

Figure 2 shows the daily shifts of the 126 fractions. The magnitudes of the online shifts were 0.41±0.36 cm, 0.60±0.42 cm, 0.41±0.30 cm in the left/right, anterior/posterior, and superior/inferior directions, respectively (Fig. 2(a)). The offline shifts were different from the online ones, with the magnitudes of 0.16±0.15 cm (p<0.05), 0.24±0.30 cm (p=0.85), and 0.30±0.24 cm (p=0.42) in the left/right, anterior/posterior, and superior/inferior directions, respectively (Fig. 2(b)). For both the online and offline shifts, the magnitude of the shifts between the first and the last seven fractions are comparable in the left/right (p=0.56 and p=0.62), anterior/posterior (p=0.57 and p=0.54) and superior/inferior (p=0.67 and p=0.59) directions.

### C. Target dose coverage and margin reductions

**Figure 3:**
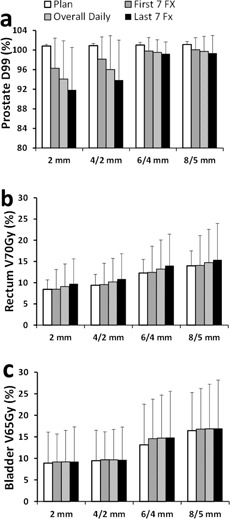
Average daily dose of the (a) prostate, (b) rectum, and (c) bladder with the clinical online IGRT shifts.

With the IMRT plans created using margins from 8/5 mm to 2 mm uniform, the average CTV (prostate) $D_{99}$ from the nine IMRT plans of each planning margin ranged from 101.1%±0.5% to 100.8%±0.3% of the prescription dose (Fig. 3(a)). The daily CTV $D_{99}$ decreased as the planning margins decreased (3, 4).

5, 6 show that the daily CTV $D_{99}$ was comparable between the 8/5 mm and 6/4 mm margins. Using the criterion of daily CTV $D_{99}>97\%$, both margins rendered sufficient target coverage in more than 90% and 98% of fractions for online and offline shifts, respectively. When the margin was reduced to 4/2 mm, the daily CTV $D_{99}$ decreased significantly. Target coverage was now only sufficient in 65.1% and 84.9% of fractions with the online and offline shifts, respectively. If a more stringent criterion of daily CTV $D_{99}>99\%$ is required, the 8/5 mm and 6/4 mm margins were sufficient in 88% and 81% fractions with the online shifts, and 97.6% and 93.4% fractions with the offline shifts, respectively.

**Figure 4:**
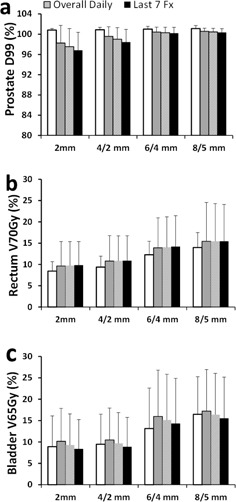
Average daily dose of the (a) prostate, (b) rectum, and (c) bladder with the offline IGRT shifts.

**Figure 5:**
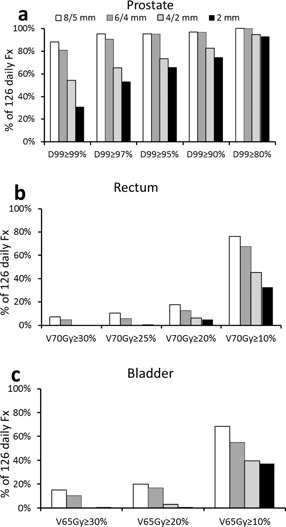
The percentage of the daily fractions that meet dose criterions for the (a) prostate, (b) rectum, and (c) bladder with the clinical online IGRT shifts.

**Figure 6:**
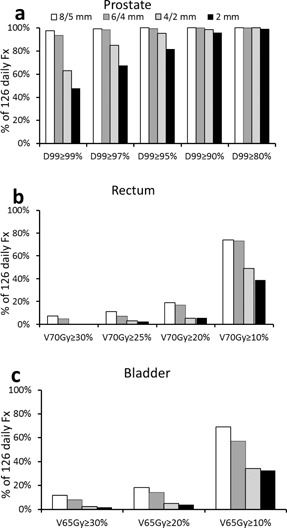
The percentage of the daily fractions that meet various dose criterions for the (a) prostate, (b) rectum, and (c) bladder with the offline IGRT shifts.

### D. Doses to organs at risk and margin reductions

The OAR doses in the original clinical plans (Figs. 3(b) and 3(c)) were well below the recommendations from the QUANTEC report (i.e., rectum $V_{70\text{Gy}}<20\%$ and bladder $V_{65\text{Gy}}<50\%$).^(14)^ As a result of the interfractional changes, the daily rectum $V_{70\text{Gy}}$ and bladder $V_{65\text{Gy}}$ were generally higher than the planned ones. The rectum $V_{70\text{Gy}}$ and bladder $V_{65\text{Gy}}$ decreased as the planning margins decreased. All fractions complied with the QUANTEC recommendation ($V_{65\text{Gy}}<50\%$) even with the 8/5 mm margin. Both rectum $V_{70\text{Gy}}$ and bladder $V_{65\text{Gy}}$ were reduced at each level of margin reduction (p<0.05). The offline shifts yielded comparable daily rectum $V_{70\text{Gy}}$ and bladder $V_{65\text{Gy}}$ to those from the online shifts (Figs. 4(b) and 4(c), and Table 1). These data confirm the dosimetric benefit of margin reduction regardless of the type (online or offline) of IGRT shifts.

**Table 1:** Dosimetric endpoints with offline IGRT shifts.

		*8/5 mm*	*6/4 mm*	*4/2 mm*	*2 mm*
			*Percent of the 126 Daily Fractions*		
Prostate (CTV) D_99_ (% of 78 Gy)	≥99%	97.6%	93.4%	62.7%	47.6%
	≥97%	99.2%	98.4%	84.9%	67.5%
	≥95%	100%	99.2%	95.2%	81.7%
	≥90%	100%	100%	98.4%	96.0%
	≥80%	100%	100%	100%	99.2%
Rectum $V_{70\text{Gy}}$	≥30%	7.1%	4.8%	0.8%	0%
	≥25%	11.1%	7.1%	3.2%	2.4%
	≥20%	19.0%	16.7%	5.6%	5.6%
	≥10%	73.8%	73.0%	49.2%	38.9%
Bladder $V_{65\text{Gy}}$	≥30%	11.9%	7.9%	2.4%	1.6%
	≥20%	18.3%	14.3%	4.8%	4.0%
	≥10%	69.0%	57.1%	34.1%	32.5%

### E. Dosimetric differences between the first seven and last seven fractions

In accordance with the greater rectum and bladder volume fluctuations, the CTV coverage of the last seven fractions was lower than that of the first seven fractions. Specifically, with the planning margins of 8/5 mm, 6/4 mm, 4/2 mm, and 2 mm uniform, the online shifts provided CTV $D_{99}>97\%$ in 93.7%, 88.9%, 50.8%, and 39.7% of the 63 fractions from the last seven days, in comparison to 96.8%, 92.1%, 79.4%, and 66.7% of the 63 fractions from the first seven days. The offline shifts provided CTV $D_{99}>97\%$ in 98.4%, 98.4%, 81.0%, and 58.7% of the last seven fractions, in comparison to 100%, 98.4%, 88.9%, and 76.2% of the first seven fractions.

The rectum $V_{70\text{Gy}}$ of the last seven fractions was higher than those of the first seven fractions for all margin sizes with the online shifts (Fig. 3(b)). The difference was not statistically significant for any margin size. Such difference was not observed with the offline shifts (Fig. 4(b)), suggesting that the difference may be due to the uncertainties in distinguishing the prostate and rectum soft tissues at online registration. The bladder $V_{65\text{Gy}}$ of the last seven fractions was comparable to those of the first seven fractions with the online shifts for all four margin sizes (Fig. 3(c)). With the offline shifts, the average bladder $V_{65\text{Gy}}$ was higher in the first seven treatment days than that in the last seven treatment days (Fig. 4(c)). The difference was not statistically significant for any margin size.

## IV. DISCUSSION

This study investigated the dosimetric effect of a series of planning margins when diagnostic quality CT‐based IGRT is used to compensate for prostate interfractional variations with translation corrections. Although the planning margins for prostate interfraction variation have been widely studied, previous publications^15^, ^16^, ^17^, ^18^ used kV‐CBCT or MVCT that displayed worse image quality than that of planning CTs. For prostate treatment, because the prostate moves independently of the pelvic bones, soft tissue alignment is necessary. However, poor soft tissue contrast in kV‐CBCT or MVCT verification images requires that implanted markers are often used as a surrogate for the prostate. The unique aspect of our study is that we used the same quality CTs for both treatment planning and IGRT, which minimized the potential variations in soft tissue‐based registration and in contour delineations for the daily verification CTs. For dosimetric evaluation of interfraction planning margins using daily verification images, accurate delineation of daily prostate contour is another challenge, particularly with poor soft tissue contrast images. Even with the diagnostic quality CTs, the interobserver variations are persistent. In our study, a single board‐certified radiation oncologist contoured all the planning CTs. Subsequently, using a patient‐specific atlas method,^(19)^ which we published previously, the same physician delineated all verification CTs to minimize the interobserver variability. The intraobserver variation for recontouring the prostate has been reported to be ∼5%.^(20)^

A few recent studies using the same diagnostic quality image‐based IGRT investigated the dosimetric impact of a single margin^13^, ^21^ or three margin sizes.^(22)^ Using 8/5 mm margin, Peng et al.^(13)^ showed a third of fractions require adaptive replanning based on daily CTV $D_{100}\geq 99\%$ of prescription dose. Using a 5 mm uniform margin, Godley et al.^(21)^ reported an accumulated CTV dose of 97.1% of the prescription dose. Wang et al.^(22)^ showed that a 5 mm uniform margin offers an average prostate $V_{95}$ of ∼97%. Other margin studies used either daily kV‐CBCT images^23^, ^24^ or implanted markers.^17^, ^25^, ^26^

Under the diagnostic quality imaging guidance, the current result showed that the residual interfractional variations still require a large margin for accommodation. This residual uncertainty may stem from: 1) not compensating for the prostate rotation; 2) prostate deformation; 3) interobserver variation in localizing the prostate during online correction. The current dose analysis based on offline shifts was performed to evaluate rotation and deformation separately from interobserver variation. With the shifts based on prostate volume contoured by the same radiation oncologist, the interobserver variation is minimized. Furthermore, offline shift data may be more relevant to SBRT scenario as direct supervision of the attending physician and physicist is required. Despite the effort of using offline image alignments with contours and using the same diagnostic quality images used for IGRT, our results still indicate the existence of residual interfractional uncertainties. Depending on the acceptable criteria of dose coverage to the prostate, the residual interfractional uncertainties still require a margin of 6/4 mm with imaging guidance and translational positioning corrections. We speculate that further margin reduction to 4/2 mm or below may require compensation of the prostate rotation and perhaps real‐time replanning to account for potential prostate deformation. Adding planning margins to the treated target is a conventional method to increase robustness of a plan, accommodating potential inter‐ and intratreatment variation. Additional analysis of our data could determine a variable margin to optimize the robustness of the plan against relative prostate motion. The proposed planning margin of 6/4 mm may be further tested on its robustness for prostate rotation and deformation when a rectal balloon is inserted.

With 3 mm planning margin, Qin et al.^(27)^ showed that the residual margin after kV‐CBCT guidance led to insufficient CTV coverage (EUD<95% of the prescription) in 18% daily fractions. It should be noted that our proposed margin does not include intrafraction motion. Different patient setup positions (supine vs. prone) may also lead to different margin requirements.^(28)^ Our findings could have important implications in prostate SBRT, as usually small margins such as 3 mm to 5 mm^29^, ^30^ or 5/3 mm^(31)^ were used. It should be noted that the target coverage obtained with the offline shifts represents the theoretical optimum scenario (or similar scenario of SBRT treatment under the direct supervision of the attending physician and physicist) because contours from planning CT and daily verification CT were used to guide imaging registration.

The application of our margin analysis to kV‐CBCT needs caution because the inferior imaging quality in kV‐CBCT may introduce additional uncertainties in localizing the prostate. With the margins of 10/6 mm and 5/3 mm, Hammoud et al.^(32)^ showed average prostate (CTV) $D_{98}$ of 97.2% and 96.7% with kV‐CBCT as the IGRT system, respectively. On the other hand, by using CT‐on‐rails for daily IGRT, our average CTV $D_{99}$ with the 6/4 mm and 4/2 mm margins were 99.2% and 96.1%, respectively (Fig. 3(a)). Direct comparison of our recommended margin with the recipe‐based interfractional margin needs to be cautious. The typical recipe‐based interfraction margin is based on a formula proposed by van Herk.^(33)^ This formula assumes that the planning margins would guarantee 90% of patients in the population receive a minimum cumulative CTV dose of at least 95% of the prescribed dose for a conventional fractionation. Using this method, even seed‐based 2D portal imaging allowed an interfractional margin as small as 4 mm.^(34)^ In the present study, we analyzed only the first seven and last seven fractions, not the entire treatment course. Our number of patients is limited. Most importantly, our analysis is based on the daily dose, not the cumulative dose. Our initial intention of the study was to use IGRT data from the conventional treatment to explore the interfractional planning margin for SBRT.

The difference in target coverage between the first seven and last seven fractions may be due to the anatomical differences. The statistical significance may be underestimated using unpaired Student's *t*‐test because the daily images of the same patient are not completely independent of each other. In our institutional experience, patients tended to develop diarrhea in the latter fractions and therefore presented larger anatomical differences from the simulation CT. The increased fluctuation of daily rectum volume in the last seven fractions may introduce more prostate rotations and deformations than those in the first seven fractions and therefore lead to decreased target coverage. The reduction in prostate CTV coverage, however, showed a weak correlation with volume changes of the prostate, rectum, and bladder. All correlation coefficients were within −0.5 to 0.5. A future study that includes the principal component analysis in the volume changes may define the correlation between the volume changes and directional‐specific margins.

The main benefit of margin reduction is to spare OAR from radiation‐induced complications. This was demonstrated dosimetrically herein as the number of daily sessions receiving high rectum or bladder dose was reduced with decrease in margin. Based on previous reports,^1^, ^2^, ^35^ rectum sparing is likely to translate into a decreased rate and level of late GI toxicity. The decrease of dose to the bladder may alleviate GU complications in the setting of standard fractionation and with prostate SBRT, which has shown higher (15.6%) late GU toxicity rate than that (12.6%) of the standard‐fractionated IMRT.^(36)^

The current feasibility of margin reduction was evaluated using daily dose profiles. The accumulated dose, while providing a straightforward endpoint, is subject to errors from the deformable image registration process.^37^, ^38^ Meeting planning target dose coverage on a daily basis, on the other hand, is likely too stringent since underdosed volume in one fraction may be compensated by other fractions in the cumulative dose analysis. In the absence of an established relationship between daily dose and accumulated dose, daily CTV $D_{99}>97\%$ was chosen as adequate coverage here based on previous studies.^17^, ^32^, ^39^, ^40^ If a strict criterion of daily CTV $D_{99}>99\%$ is used, even the 8/5 mm margin provides only 88% fractions with adequate target coverage (Table 2).

The current margin analysis did not account for intrafractional variation. Our clinical practice has a typical beam‐on time of 7 min. Based on previous studies with similar treatment time and patient setup,^40^, ^41^ a 3 mm uniform margin for the intrafractional motions may suffice for our clinical practice. Since the combined effect of intrafractional and interfractional variations is not a simple addition, the whole PTV margin for the composite effect may be accurately estimated only when both types of variations are captured in future studies.

**Table 2:** Dosimetric endpoints with online IGRT shifts.

		*8/5 mm*	*6/4 mm*	*4/2 mm*	*2 mm*
			*Percent of the 126 Daily Fractions*		
Prostate (CTV) D_99_ (% of 78 Gy)	≥99%	88.1%	81.0%	54.0%	31.0%
	≥97%	95.2%	90.5%	65.1%	53.2%
	≥95%	95.2%	95.2%	73.0%	65.9%
	≥90%	96.8%	96.8%	82.5%	74.6%
	≥80%	100%	100%	94.4%	92.9%
Rectum $V_{70\text{Gy}}$	≥30%	6.3%	1.6%	0%	0%
	≥25%	10.3%	5.6%	0.8%	0.8%
	≥20%	17.6%	12.7%	6.3%	4.8%
	≥10%	76.2%	67.5%	45.2%	32.5%
Bladder $V_{65\text{Gy}}$	≥30%	15.1%	10.3%	0.8%	0.8%
	≥20%	19.8%	16.7%	3.2%	0.8%
	≥10%	68.3%	54.8%	39.7%	37.3%

## V. CONCLUSION

With daily diagnostic quality imaging guidance and translation position correction, the interfractional planning margin may be reduced from 8/5 mm to 6/4 mm. Further reduction of the margin to 4/2 mm or less may result in inadequate dose delivered to the prostate unless compensation for prostate rotation and deformation is incorporated.

## COPYRIGHT

This work is licensed under a Creative Commons Attribution 4.0 International License.

## Supporting information

Supplementary Material
