# Supplementary figures and images for: Using daily diagnostic quality images to validate planning margins for prostate interfractional variations
Source: J Appl Clin Med Phys. 2016 May 8;17(3):61–74. doi: 10.1120/jacmp.v17i3.5923 (PMC5690910; doi:10.1120/jacmp.v17i3.5923)

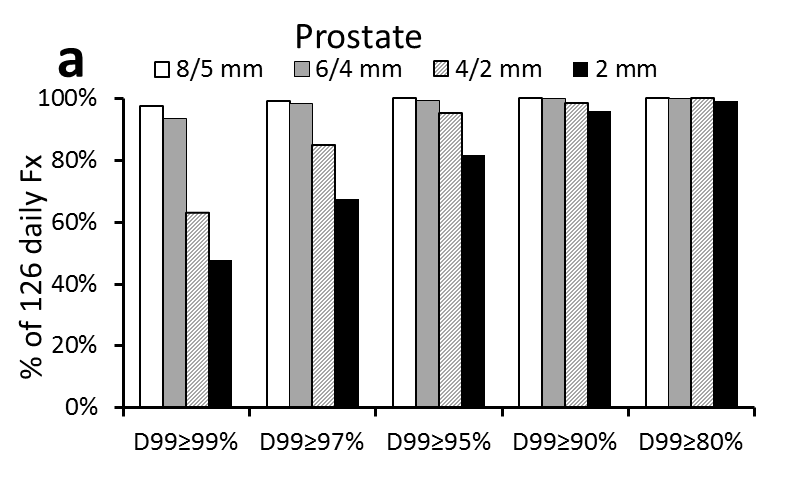

Supplement: Supplementary file 1 — Supplementary Material [file ACM2-17-061-s001.tif]
